# Design of low-threshold photonic-crystal surface-emitting lasers with confined gain regions by using selective area intermixing

**DOI:** 10.1186/s11671-023-03911-8

**Published:** 2023-10-30

**Authors:** Chia-Jui Chang, Lih-Ren Chen, Kuo-Bin Hong, Tien-Chang Lu

**Affiliations:** 1 Department of Photonics, College of Electrical and Computer Engineering, National Yang Ming Chiao Tung University, Hsinchu City, 30010 Taiwan; 2 Semiconductor Research Center, Hon Hai Research Institute, Taipei City, 23678 Taiwan

**Keywords:** Photonic-crystal surface-emitting laser, Low threshold, Small divergence angle, Quantum-well intermixing

## Abstract

Photonic-crystal surface-emitting lasers have many promising properties over traditional semiconductor lasers and are regarded as the next-generation laser sources. However, the minimum achievable lasing threshold of PCSELs is still several times larger than that of VCSELs, and limiting its applications especially if the required power is small. Here, we propose a new design that reduces the gain region in the lateral plane by using selective quantum-well intermixing to reduce the threshold current of PCSELs. By performing theoretical calculations, we confirmed that the threshold current can be lowered by a factor of two to three while keeping the PCSEL’s advantage of small divergence angle.

**Supplementary Information:**

The online version contains supplementary material available at 10.1186/s11671-023-03911-8.

## Introduction

In the last two decades, photonic-crystal surface-emitting lasers (PCSELs) have attracted significant attention due to their promising functionalities and improved performance compared with traditional semiconductor lasers [1–15]. PCSELs are a type of semiconductor laser that has a waveguide structure that confines the light in the vertical direction, similar to other edge-emitting lasers, but with a photonic crystal (PC) layer embedded in their structure. To achieve surface emission, the reciprocal lattice vector of the photonic crystal is set to be the same magnitude as the wave vector of the designed wavelength. By utilizing second-order Bragg diffraction, the waves in the waveguide can couple with each other and oscillate in the horizontal plane, similar to the distributed-feedback laser (DFB laser), and part of the power is scattered to the vertical direction due to first-order diffraction. With the unique in-plane oscillation and out-of-plane emission architecture, PCSELs have power scalability with device size while retaining single-mode lasing and narrowing the divergence angle accordingly. Many efforts have been made to increase the output power [9–15]. Recently, 29-Watt continuous-wave operation of PCSELs with a 2 mm diameter was demonstrated, which had a divergence angle as small as 0.4 degree [9]. Even higher kW operation has also been theoretically predicted [13]. Furthermore, many functionalities such as beam steering, vector beam generation, polarization control, and various beam patterns have been achieved by directly modulating the photonic crystal structure [3–8], paving the way for the next generation of ultracompact light sources.

However, for some applications such as bio-stimulation, environmental sensing, and short-distance detection, only a laser power of several mW with low operation current is required. In this regard, the lasing threshold current becomes an important factor for these applications. Vertical-cavity surface-emitting lasers (VCSELs) have been applied in these applications for many years. However, the relatively large divergence angle of VCSELs typically requires collimating lenses to efficiently couple the laser light out. On the other hand, PCSELs that have a relatively small divergence angle typically require a diameter larger than 30 µm to achieve sufficient in-plane feedback [16, 17], which is 2–3 times larger than the typical emission apertures of VCSELs. The threshold current is squarely proportional to the diameter if we assume similar modal losses. To suppress the lasing threshold in PCSELs, in-plane photonic heterostructures have been proposed [17]. In this method, the lasing threshold of PCSELs is suppressed with additional feedback heterostructure, reducing the required device area. However, the corresponding divergence angle is inevitably broadened, and the gain margin is narrowed. Another proposed method utilizes Fano resonance to reduce the radiation loss [18]. The lasing area is maintained to be large but has poor light extraction efficiency.

Here, we propose a new design that can effectively reduce the threshold current of PCSEL by restricting the current injection gain region while maintaining the low divergence emission angle by extending the photonic crystal out of the gain region. In order to eliminate the absorption loss from out of the gain region, selective quantum-well intermixing (QWI) method can be applied on PCSELs. QWI is well established as a relatively simple and efficient method for defining passive sections in semiconductors [19–25]. In passive sections, QW and barrier compositions are intermixed, and thus higher bandgap energy and lower interband absorption can be realized. Depending on the methods, we can even make passive region be with low conductivity and suppress the current flowing through here [21, 24, 25]. By theoretical calculation, we investigate the threshold current, slope efficiency (SE), and power conversion efficiency (PCE) dependence on the injection region radius. We have proved that this new design is able to fulfill the desired functions of low operation current and low divergence angle at the same time.

## Structure and fabrication method

**Fig. 1:**
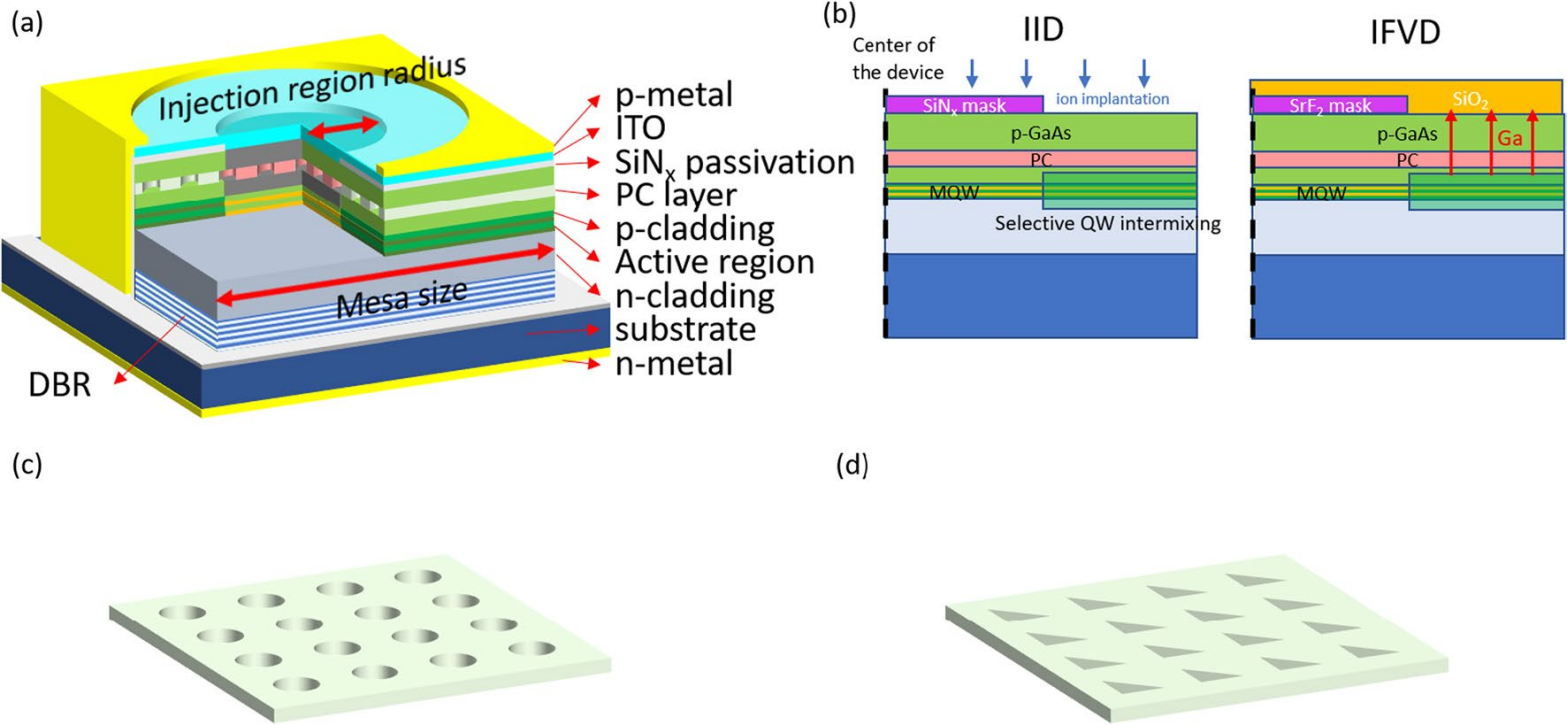
**a** Schematic diagram of PCSEL structure with confined injection region. **b** Probable fabrication processes of selective QWI. Arrangement of PCSEL with **c** square lattice with circular (CC) and **d** right-angled isosceles triangle (RIT) air hole

We choose the PCSELs with 940 nm lasing wavelength, which is grown with InAlGaAs-based materials for the following analysis. The PCSEL structure is shown in Fig. 1(a), and the thickness of each layer is shown in Table 1. The active region and separation confinement heterostructure (SCH) layers (not labeled in the figure) are sandwiched in the two cladding layers to form the waveguide structure. The PC layer is constructed with a periodic pattern featuring a period of 280 nm, which corresponds to the effective index of 3.35 for the fundamental mode within our waveguide structure. The PC layer is embedded in the waveguide layer with tuned distance from active region. We use a DBR structure below the waveguide as backside reflector to increase the output efficiency. Above the epitaxial layer, SiN_x_ pattern is used to define injection region, which is smaller than the PC region, and we assume that there’s nearly zero carrier injection for active layers under SiN_x_. P-metal layer is for the contact and an ITO layer, served as transparent conduction layer, is deposited between the metal and epilayer in order to achieve good uniformity of current injection [15]. The mesa structure is etched to substrate for isolation, and the PC region is assumed to have the same size with device mesa. The detailed design and optimization of PCSEL structure can refer to our previous publications [14, 26]. For those regions out of the current injected region, strong absorption of MQWs will seriously increase the threshold gain of PCSEL. Here, we propose that QWI method can be adopted to reduce the interband absorption. Schematic diagram of the two usually adopted method, impurity-induced disordering (IID) and impurity-free vacancy disordering (IFVD), is shown in Fig. 1b. In IID method, the interdiffusion is activated with impurity insertion. The impurities are inserted using either implantation or diffusion, and the used impurities are usually Si, Ge, S, Se. The selected intermixing region can be defined with a mask, which can hinder implantation. In IFVD method, the interdiffusion is activated with group-III vacancies instead of impurity insertion. A SiO_2_ layer is capped on the epitaxial layer and again selected region can be defined with a mask. The Ga atom is known to be soluble to oxide, so the region contacted with oxidation layer undergoes intermixing process. The two methods have their own advantages and drawbacks. Depending on what impurity is used, IID method can selectively tune the conduction property to achieve better injection control, but the impurity-induced loss may also increase the lasing threshold [21, 25]. Different from IID method, IFVD method results in better crystalline property, but its tunability on the conductivity is limited. The QWI can be implemented either before or after the PC layer regrowth.

**Table 1:** Structure of the calculated PCSEL device

Layer	Thickness (nm)
Substrate (GaAs)	
DBR (Al_0.8_GaAs/Al_0.2_GaAs)	(76/68) × 15
n-clad (Al_0.46_GaAs)	2000
n-SCH (GaAs)	60
Active (In_0.13_GaAs/GaAs)	(8/12) × 3
p-EBL (Al_0.46_GaAs)	3.29
PC (Air/GaAs)	250
p-clad 2 (Al_0.46_GaAs)	2000
p-contact (GaAs)	100

## Simulation method

To consider a periodic structure with finite size properly, we first construct the 3D coupled-wave theory (CWT) model and focus our analysis on modes with TE-like polarization, i.e., electric field is predominantly transverse to the interface of epilayers [27–29]. This model provides a fast yet precise estimation of the field distribution of four fundamental waves propagating in four directions (+ x, − x, + y, -y), each of which has a slowly varying envelope function denoted as *R*_*x*_(x, y), *S*_*x*_(x, y), *R*_*y*_(x, y), and *S*_*y*_(x, y). The coupling behavior among four basic waves, high-order waves, and radiative waves can be simply described with a set of coupled-wave equations, in which the coupling matrix **C** describing the coupling strength among those waves is derived from epitaxial structure and geometry of the PC layer. The derivation of the coupling matrix is detailed in Ref. 27. The set of coupled-wave equations is an eigenvalue problem with an eigen value equal to α − iδ, where α denotes the threshold gain and δ denotes the detuning of wavevector from Bragg condition, and the eigenvector equal to the field distribution of the envelop of the four basic waves. To study the impact of injection region radius on laser characteristics, a non-uniform gain distribution with only propagation loss, α_propag_, outside the injection region needs to be applied. The propagation loss here refers to the power loss per unit length as it travels along the waveguide. This loss can be quantified as 13.95 cm^−1^, which is derived from the imaginary part of the propagation constant. The value is consistent with variable stripe length measurements obtained from edge-emitting lasers with similar waveguide structures [30]. We aim to model a structure where gain is confined to the injection region, while there's no net gain in QW outside the region. To achieve this, we introduce additional loss terms into the field derivatives of CWT model. This enhances loss where needed, such as $\frac{\partial {R}_{x}}{\partial x}=\left(\alpha -i\delta \right)Rx+i{C}_{11}{R}_{x}+i{C}_{12}{S}_{x}+i{C}_{13}{R}_{y}+i{C}_{14}{S}_{y} (original terms)-{\alpha }_{ext}{R}_{x}$, to the four fields. When we introduce this additional loss, α_ext_, outside the injection region, it results in an increase in the derived lasing threshold, denoted as α. Under lasing conditions, the propagation loss outside the injection region is α_ext_ − α. Our objective is to find the value of α_ext_ at which α_ext_ − α equals to α_propag_, indicating that only α_propag_ outside the injection region. This is achieved through an iterative process. Importantly, the rate of α's growth is intentionally slower than that of α_ext_, ensuring that the condition α_ext_ − α = α_propag_ is met as α_ext_ reaches a certain threshold.

In this paper, our main purpose is to deliver a method for reducing threshold current. We do not take spatial hole burning (SHB) and refractive index change into account, the more accurate model may require the use of time-dependent CWT model [31]. However, the impact of SHB is expected to be minimal due to the uniform field distribution in the proposed structure's injection region. Additionally, the refractive index change due to nonuniform injection is thought to have positive effect on threshold as discussed in Ref. [31]. As the lasing usually happen at band edge of concave downward, the lower carrier density outside the injection region can result in a downshift of the band edge, leading to a forbidden bandgap to the lasing mode in the center.

In the following section, we analysis the PCSELs having square lattice with circular (CC) and right-angled isosceles triangle (RIT) air hole in basis as shown in Fig. 1c and d, respectively, for comparison. PCSELs with CC hole, having a symmetric unit cell, are thought to have lower radiation loss because of the destructive interference at far field [9, 14]. On the other hand, PCSELs with RIT hole, which break the destructive interference at far field, are able to realize higher laser output. For both cases, we consider mesa sizes of 100 and 140 µm, and the injection region radius ranges from 20 to 40 µm and compare these results with unmodified structure. Detailed calculation parameters are listed in Table 2. [35–37]

**Table 2:** Structural and calculation parameters

Parameter	Value
Al_x_GaAs refractive index	3.55–0.5x
Filling factor of photonic crystal	0.25
QW confinement factor	10.5%
Propagation loss	13.95 cm^−1^
Transparency carrier density	1.0 × 10^18^ cm^−3^
Differential gain	3.0 × 10^–15^ cm^2^
Saturated gain	5800 cm^−1^
Gain compression factor	2.0 × 10^–17^ cm^3^
Spontaneous recombination coefficient	A = 1.0 × 10^5^ s^−1^ B = 1.4 × 10^–10^ cm^3^ sec^−1^ C = 8.1 × 10^–29^ cm^6^ sec^−1^
Spontaneous emission factor	1.0 × 10^−4^
Current injection efficiency	0.9

## Results and discussion

**Fig. 2:**
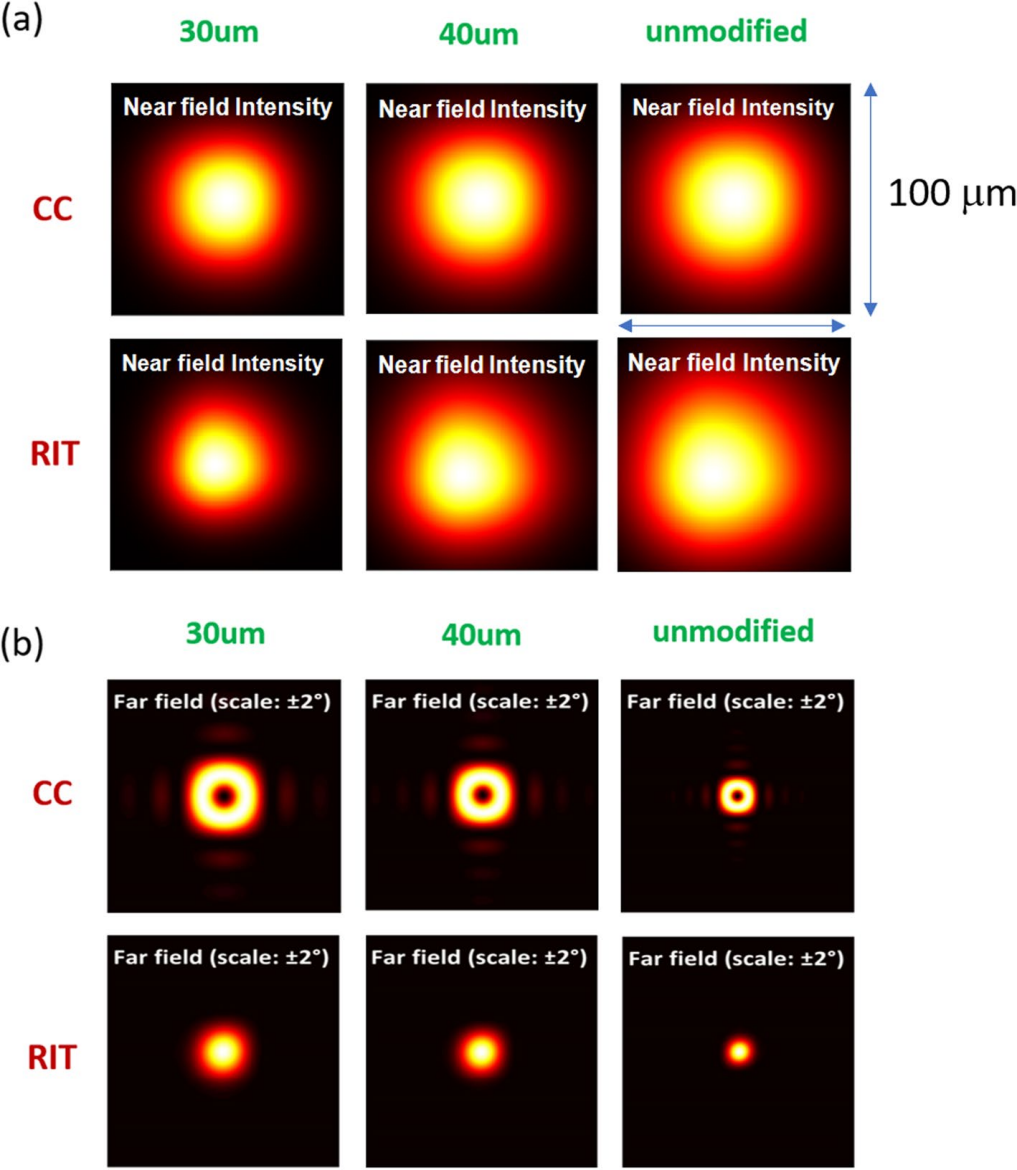
**a** NFP and **b** FFP of PCSELs with a mesa size of 100 μm and different injection region radius. The green text corresponds to the injection radius of the PCSELs

Figure 2a shows the simulation results of the near field patterns (NFPs) of PCSELs with mesa size of 100 µm. The near-field intensity exhibits a fast decay outside the injection radius. In PCSELs with smaller injection radius, the NFPs shrink correspondingly. The full-width at half maximum (FWHM) of NFPs narrows from 60 to 42 µm as the injection radius decreases from unmodified structure to 30 µm in the CC case. The NFPs of PCSELs with CC and RIT holes are similar in size, but those with RIT holes deviated a little from the center of the device, manifesting the unequal backscattering strength in positive and negative propagation direction. The corresponding far-field patterns (FFPs) are shown in Fig. 2b. Doughnut-shaped FFPs in PCSELs with CC holes are resulting from the destructive interference of anti-symmetric field at gamma point, while they are solid in the center in RIT cases, reflecting the breaking of symmetry. As the injection region radius decreases, the divergence angle increases from approximately 0.6 to 0.9 degrees. However, even with this increase, the divergence angle remains significantly smaller than the 5-to-10-degree divergence angles typically observed in single-mode VCSELs [32, 33].

**Fig. 3:**
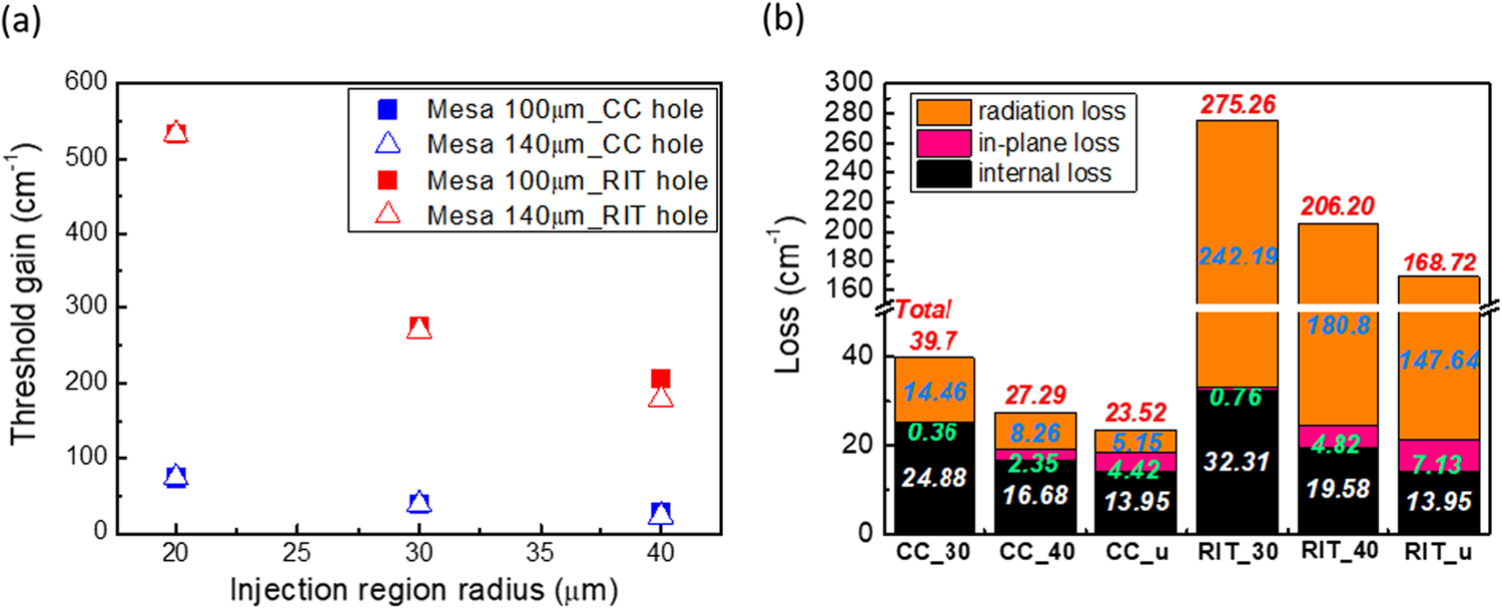
**a** Threshold gain of PCSELs with different photonic crystal air hole and mesa size (CC/RIT, mesa size 100/140 mm) versus injection region radius and **b** the optical loss component of PCSELs with mesa size 100 mm. The x label represents their air hole shape and injection region radius (in µm), where “u” stands for unmodified structure

The threshold gain of PCSELs with varying injection radius is presented in Fig. 3a. First, the threshold gain of PCSEL with CC hole is below 100 cm^−1^, while it is above 200 cm^−1^ for that of RIT hole. The threshold gain of the PCSEL with CC hole is considerably lower than that of the RIT hole owing to destructive interference in the far field. Second, the threshold gain of both types of air holes in the PC increases as the injection radius decreases, as expected. Furthermore, we noted that the threshold gain of PCSELs with a mesa size of 100 µm and 140 µm tends to converge as the injection region becomes much smaller compared with the mesa size. Under this condition, the electric field is well-confined in the center of the structure, lowering the effect of device size.

Following, we investigate the loss component of the oscillation modes. The loss component of an optical mode can be decomposed into three distinct components:

1 $$\alpha = \alpha_{i} + \alpha_{rad} + \alpha_{in - plane}$$

where they are defined as internal loss, radiation loss, and in-plane loss. Internal loss refers to the effective loss as light propagates unit length in the waveguide. While similar to the propagation loss mentioned in the simulation method, internal loss may deviate from the propagation loss, especially as the injection region shrinks. In contrast, radiation loss describes the power that is emitted from the top and bottom surfaces of the waveguide and is closely related to the laser's output power. Maximizing the contribution of radiation loss is critical for achieving high output efficiency. Finally, in-plane loss quantifies the amount of power that is radiated from the side walls of the mesa structure. The three components of loss can be extracted from the derived fields and optical constants [27]. Figure 3b shows the loss component of PCSELs with mesa size of 100 µm. First, we compare the results between CC and RIT case. The radiation loss of the RIT case is significantly higher than that of the CC case and represents the primary source of optical loss. Therefore, PCSELs with RIT hole have much larger SE at the cost of larger threshold. In-plane loss of the RIT case is also larger than that of the CC case, consistent with the previous results that the NFPs of the RIT case is larger and deviate a little from the center. We now turn to the impact of the injection region radius on the PCSEL performance. As the radius decreases, the electric field is more tightly confined in the center of the device, leading to lower in-plane loss. Conversely, the internal loss and radiation loss increase as the injection region radius decreases. The proposed structure is shown to have beneficial impact on suppressing the in-plane loss.

**Fig. 4:**
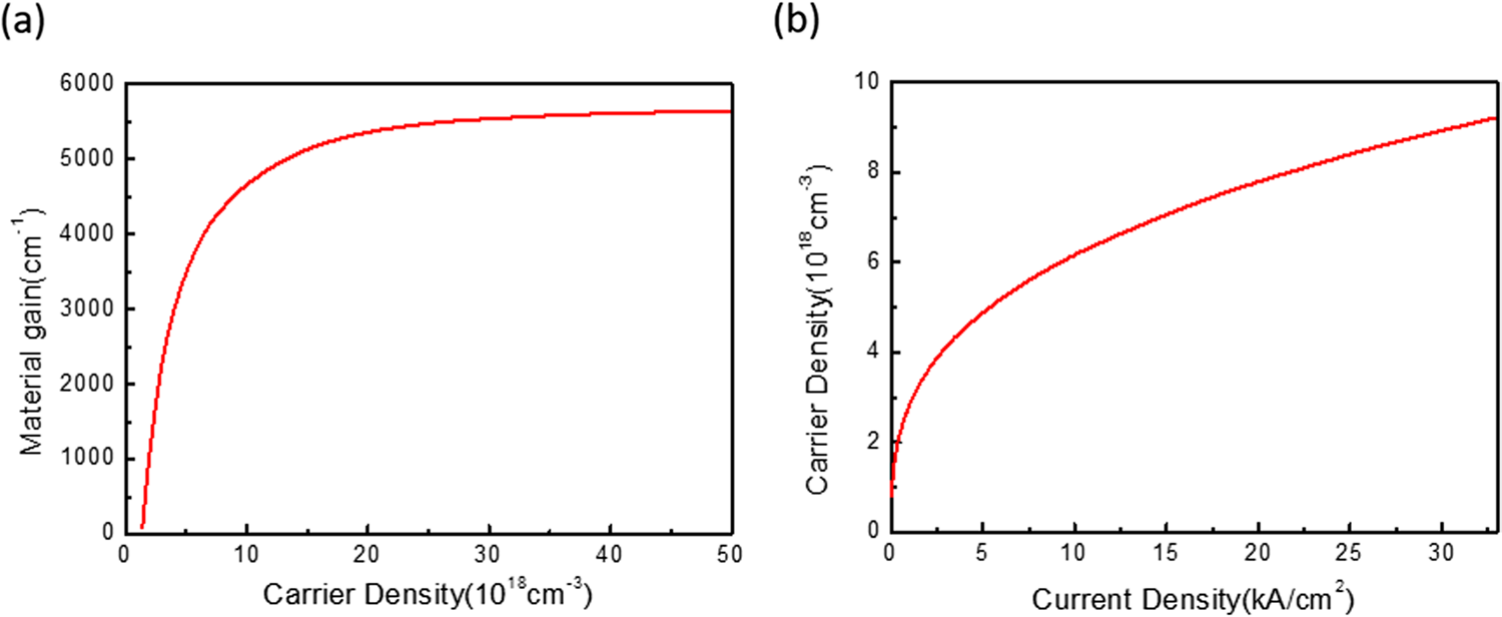
**a** The material gain of InGaAs QW as a function of carrier density. **b** Carrier density inside the QW as a function of injection current density

**Fig. 5:**
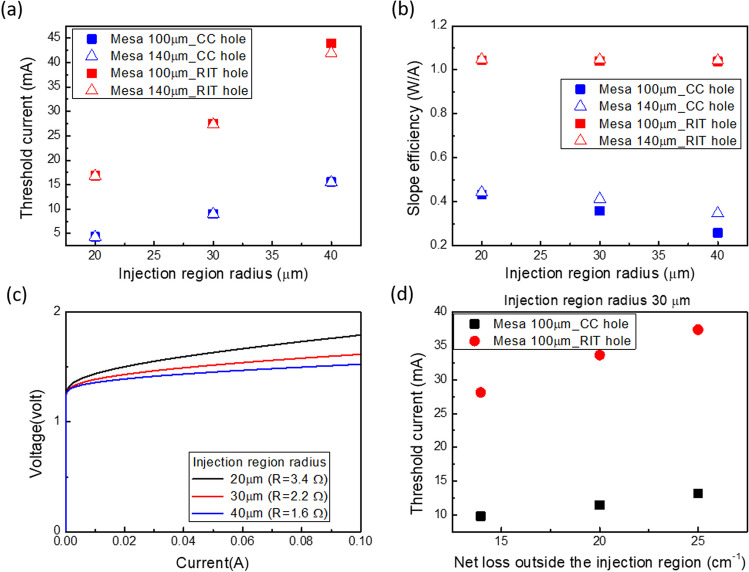
**a** Threshold current and **b** Slope efficiency of PCSELs with different mesa size and air hole shape versus injection radius. **c** I–V curve of devices with different injection region radius. **d** Threshold current of PCSELs with mesa size of 100 µm and injection region radius of 30 µm, as a function of net loss outside the injection region

Once we have ascertained the threshold gain using the coupled-wave method, we can then calculate the threshold current by combining two relationships: the material gain of InGaAs QW versus carrier density within the QW, and the carrier density within the QW versus injection current density. These curves have been obtained using the PICS3D software and are shown in Fig. 4a and b. The threshold current obtained from the threshold gain is shown in Fig. 5a**.** The threshold current of PCSELs with RIT holes is approximately three times that of CC holes, despite the threshold gain being approximately seven times higher. On the other hand, the threshold current significantly decreases as the injection region radius shrinks. For instance, it drops from 75 mA for the unmodified structure to 17 mA for an injection region radius of 20 µm in the RIT case. In the CC case, the threshold current is also suppressed from 25 mA of unmodified structure to below 5 mA, which is close to the threshold current of commercial VCSELs with diameters of about 10 µm. We obtain their SE by assuming 90% of injection efficiency and a nearly 100% reflectivity at the bottom side of the devices, that is, almost all the light can radiate from the top surface. The derived SE is shown in Fig. 5b. Despite having a much higher threshold current, PCSELs with RIT holes exhibit a much higher SE due to much larger radiation loss, which can reach 1.04 W/A in PCSELs with RIT hole, while it is only 0.26– 0.43 W/A for CC hole. We observed that the SE does not vary significantly with the injection radius in the RIT case. However, in the CC case, SE is greatly improved with smaller injection region, due to the relatively large ratio of in-plane loss of unmodified structure. We also calculate their corresponding I–V curve according to their injection radius with commercial finite element analysis based on drift–diffusion model and Poisson equation. Figure 5c shows the results for injection radius of 20, 30, and 40 µm when the mesa size is 100 µm. Their turn on voltage is at about 1.45 V, and serial resistance is increased from 1.6 to 3.4 Ω as the injection region radius decreased. Furthermore, we have extended our investigation by calculating the threshold current as a function of net loss outside the injection region. This allows us to simulate scenarios where residual loss exists in the intermixed quantum well (QW) regions. To achieve this purpose, we introduced additional loss into the propagation loss, as mentioned in our simulation method. For this specific analysis, we consider solely of PCSELs with a mesa size of 100 μm and an injection region radius of 30 μm. The results of these calculations are presented in Fig. 5d, which illustrates how the impact of threshold reduction is diminished in the presence of residual loss.

**Fig. 6:**
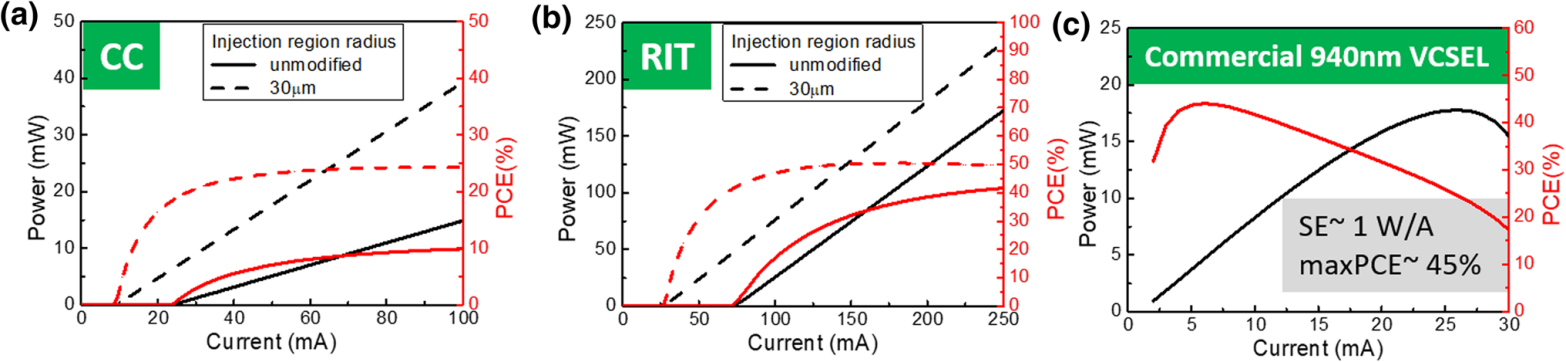
Power and PCE versus current of **a** CC hole and **b** RIT hole PCSELs and **c** commercial VCSEL with a 10 µm oxide aperture

Figure 6a and b illustrates the power and power conversion efficiency (PCE) of PCSELs with a mesa size of 100 µm and injection region radius of 30 µm, as well as the unmodified structure. The proposed structure results in a significantly smaller threshold current and slightly improved slope efficiency, leading to a higher PCE compared to the unmodified structure. However, due to poor output efficiency, PCSELs with CC holes have a PCE that does not exceed 25%. In contrast, PCSELs with RIT holes can achieve a high PCE of 51%. Compared with the measurement results of commercial 940 nm single-chip VCSEL shown in Fig. 6c with a 10 µm oxide aperture, PCSELs with CC hole have a relatively higher threshold and lower SE. But it is worth noting, the main cause of thermal rollover effect in the L-I curve of VCSEL is thought to be the higher serial resistance and poor heat dissipation caused from the oxidation confinement aperture. The thermal resistance of PCSELs is thought to be relatively smaller and the thermal rollover effect may be alleviated. Furthermore, PCSELs have many good characteristics over VCSELs such as small divergence angle, it would be acceptable at the cost of some power efficiency loss when considering overall coupling efficiency. Despite the threshold of PCSELs of RIT hole is still much larger than VCSELs, the theoretically predicted high PCE is promising, and the method proposed here can be combined with others to achieve even lower threshold. With characteristics of small divergence angle, low threshold current, and good output efficiency, we believe this new design of PCSELs with confined gain region can be very promising in sensing and short-distance detection applications. Furthermore, considering the challenges of commercializing long wavelength VCSELs due to the difficulties in making InP-based distributed Bragg reflectors [34], long wavelength InP-based PCSELs with this new design can become excellent alternatives in realizing long wavelength coherent emitters for bio-sensing applications requiring small operation current and small divergence angle.

## Conclusions

We propose a new method, which applies reduced gain region by utilizing the selective QWI method to reduce the threshold current of PCSELs and perform a theoretical calculation to optimize the injection radius of the proposed structure. The optimized threshold current in PCSELs with CC hole is below 5 mA, which is very close to the commercial VCSELs with a 10 µm oxide aperture. On the other hand, the optimized threshold current in PCSELs with RIT hole is about 20–45 mA, which is still large if compared with VCSELs. However, their PCE can reach 51% and maintain a high value until several hundred mA because of small thermal resistance. This work shrinks the gap of threshold current between PCSELs and VCSELs and is able to combine with other proposed method that paves the way for next-generation laser sources for emerging applications.

### Supplementary Information

**Additional file 1. **Supporting Information.

## Data Availability

Data underlying the results presented in this paper are not publicly available at this time but may be obtained from the authors upon reasonable request.

## Code availability

Source code is available upon request from the corresponding author.
