# Supplementary material for: Design of low-threshold photonic-crystal surface-emitting lasers with confined gain regions by using selective area intermixing
Source: Discov Nano. 2023 Oct 30;18(1):134. doi: 10.1186/s11671-023-03911-8 (PMC10616058; doi:10.1186/s11671-023-03911-8)
Supplement: Supplementary file 1 — Additional file 1. Supporting Information. [file 11671_2023_3911_MOESM1_ESM.docx]

**Supporting Information**

**Design of low threshold photonic-crystal surface-emitting lasers with confined gain regions by using selective area intermixing**

Chia-Jui Chang^1^, Yu-Wen Chen^1^, Lih-Ren Chen^1^, Kuo-Bin Hong^2^, Jhih-Sheng Wu^1^, Yao-Wei Huang^1^ and Tien-Chang Lu^1*^

Chia-Jui Chang^a, c^, Lih-Ren Chen^a, d^, Kuo-Bin Hong^b, e^, and *Tien-Chang Lu^a, f^

^a^Department of Photonics, College of Electrical and Computer Engineering, National Yang Ming Chiao Tung University, Hsinchu City 30010, Taiwan

^b^Semiconductor Research Center, Hon Hai Research Institute, Taipei City 23678, Taiwan

^c^blue12480315.ee08@nycu.edu.tw, ^d^albertchen.eo07g@nctu.edu.tw, ^e^robin.kb.hong@foxconn.com, ^f^timtclu@nycu.edu.tw

*Corresponding author: Tien-Chang Lu

1. **Simulation details**

1.1 boundary condition

As the oscillation area of our PCSEL device is defined by the mesa, we assume an air-surrounded boundary condition in the simulation model. The boundary condition can be derived using the Fresnel equations under conditions of normal incidence. In this work, the reflection coefficient r is given by the formula: r = (n_1_-n_2_)/(n_1_+n_2_), where n₁ represents the effective refractive index of the PCSEL waveguide structure with a value of 3.36, and n₂ is the refractive index of the air, which is equal to 1. More specifically, we express the electric field of the two fundamental waves propagating in +x and -x direction as *Rx*(*x, y*)Θ0(*z*)exp(-iβ_0_x) and *Ey,−*1*,*0 = *Sx*(*x, y*)Θ0(*z*)exp(iβ_0_x), where Θ_0_ represents the electric field distribution of fundamental waves in z- direction. The boundary condition for *R*_x_ and *S*_x_ at the left boundary where x = -L/2 is then *R*_x_exp(iβ_0_L/2) = r×*S*_x_exp(-iβ_0_L/2). The boundary conditions at the other 3 sides can also be obtained with the same way.

1.2 Calculation of coupling coefficients (coupling matrix **C**)

The matrix C was computed following the cited paper Ref. 27. In this paper, C is composed of 3 parts, i.e., **C**_1D_, **C**_2D_, and **C**_rad_, which correspond to direct 180-degree back-scattering, indirect coupling through high-order modes (Referring to waves with an in-plane k-vector represented as (mβ_0_, nβ_0_), where m and n satisfy the condition $\sqrt{m^{2}+n^{2}}>1$), and indirect coupling through radiation wave (m = n = 0).

In the calculation of **C**_2D_ and **C**_rad_, the electric field distribution of higher-order waves and radiation waves is necessary to count the strength of the coupling through them. It is worth noting that we employ different methods for calculating the field distribution of higher-order waves and radiation waves. In the cited reference Ref. 27, they adopted green’s function method for this purpose. The green’s function here refers to the solution of *f*(z) of the equation: $\left[ \frac{\partial^{2}}{\partial z^{2}}+k_{0}^{2}n_{0}^{2}\left( z \right)-\left( m^{2}+n^{2} \right)\beta_{0}^{2} \right]f\left( z \right)=\delta(z-z^{'})$. The solution consists of two distinct homogeneous solutions on either side of z = z', and they exhibit a discontinuity in their derivatives, precisely a jump of magnitude 1.

In the calculation of C_rad_, where m = n = 0, the formulas on either side are $Ae^{ik_{0}n_{0}z}+Be^{-ik_{0}n_{0}z}$and $Ce^{ik_{0}n_{0}z}+De^{-ik_{0}n_{0}z}$, respectively. The constrains which determine the coefficient A~D are: (1) discontinuity in their derivatives which equal to 1 at z = z' and (2) they are continuous at z = z'. The other two constrains are the boundary conditions at the two boundaries. The formula used in Ref. 27: $G\left( z,z^{'} \right)=-\frac{i}{2k_{0}n_{0}}e^{-ik_{0}n_{0}|z-z^{'}|}$ is somewhat overgeneralized, and not suitable especially when the reflection of radiation wave from n-DBR matters. To achieve the accurate distribution of radiation waves and higher-order waves, we employ the mathematical software to directly solve equations A1 and A2. Herein, we present the coupling coefficients obtained for CC (FF=0.25) and RIT (FF=0.25), quantified in units of cm^-1^, for the purpose of reference.

$$\begin{matrix} \text{C}_{CC}\text{=}\left[\begin{matrix} 732+60i & 658+60i & 507+0i & -507+0i \\ 658+60i & 732+60i & -507+0i & 507+0i \\ 507+0i & -507+0i & 732+60i & 658+60i \\ -507+0i & 507+0i & 658+60i & 732+60i \end{matrix}\right] \\ \text{C}_{\text{RIT}}\text{=}\left[\begin{matrix} \text{745 + 44}i & 256-777i & 194+0i & -81+310i \\ 232+863i & \text{745 + 44}i & -81-310i & 194+0i \\ 194+0i & -81+310i & \text{745 + 44}i & 256-777i \\ -81-310i & 194+0i & 232+863i & \text{745 + 44}i \end{matrix}\right] \end{matrix}$$

1.3 Discretization

To prevent the emergence of undesirable checkerboard patterns in our solutions, we have adopted an arrangement in which the fields and their derivatives are discretized at identical spatial positions within each equation. The schematic diagram below illustrates how the four fields in equations are discretized. Each equation is located at the center of each grid (hollow circle), and the field is obtained with the average of the two adjacent field point, while the derivative is their difference divided by Δx or Δy. For example, in the equations at p_2_ point, each Rx is replaced with (R_x,1,1_ + R_x,1,2_)/2, while $\frac{\partial R_{x}}{\partial x}$ is replaced with (R_x,1,2_ - R_x,1,1_)/Δx. Finally, it's also worth noting that R_x_ doesn't appear at the left-side boundary because it's not an independent variable; instead, it's related to S_x_ through the previously mentioned boundary condition.
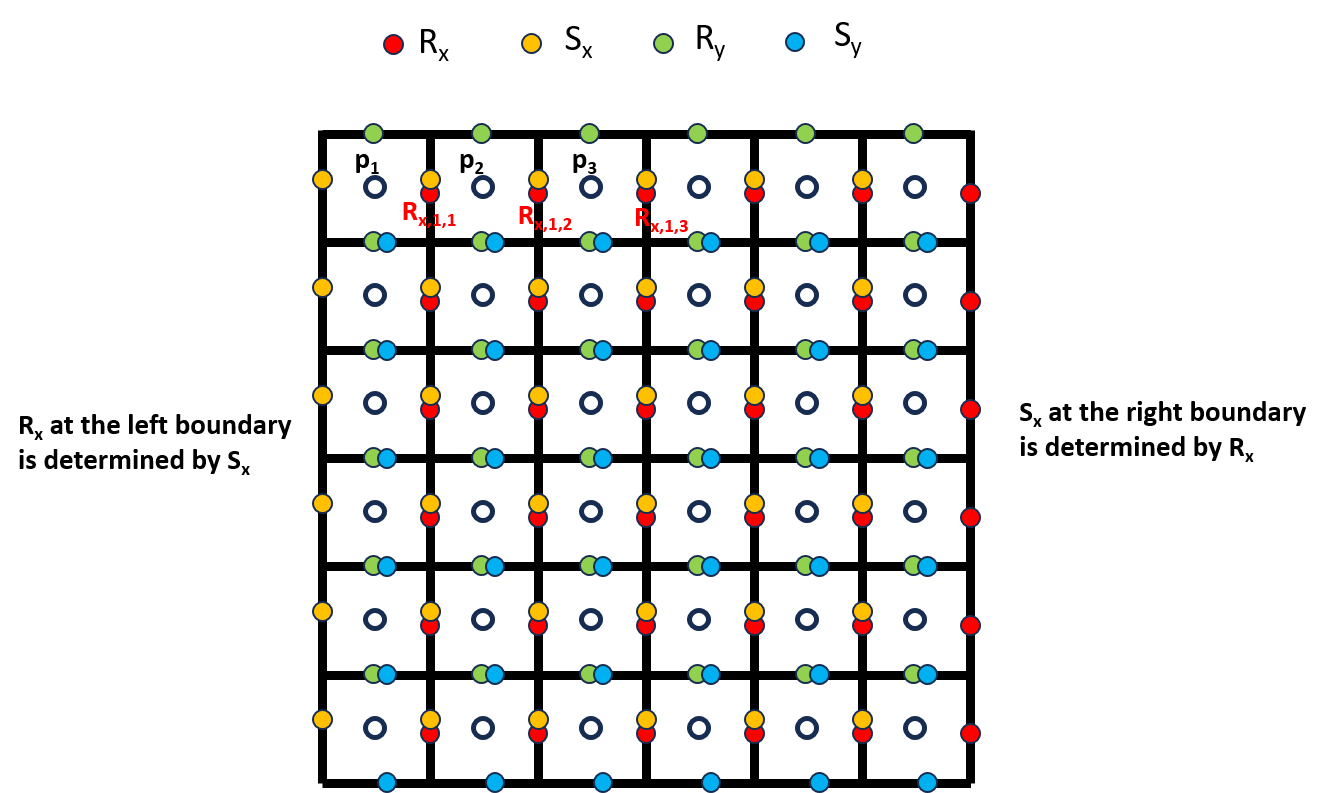


**Fig. S1 | Discretization of the fields.** The figure shows how the four fields *R*_x_, *S*_x_, *R*_y_, *S*_y_ are discretized in our simulation. The hollow circles denote the respective positions of each equation within the discretization.

1.4 Far field pattern calculation

The far field is calculated from the radiation wave ΔE_x_(x, y, z) and ΔE_y_(x, y, z) at near field by adopting Fraunhofer diffraction equation. More specifically, once the four fundamental wave fields are determined, we can extract both the x and y components of the radiation field distribution in the near field. The x and y components of the far field are calculated individually and subsequently combined as follows: I_FF_(θ_x_, θ_y_) = abs(E_x,FF_^2^(θ_x_, θ_y_) + E_y,FF_^2^(θ_x_, θ_y_)). The radiation field selected for calculating the far-field pattern is chosen at a vertical position slightly above the PC layer.
